# How stable isotopes can advance nutrition assessments to inform sustainable food systems

**DOI:** 10.1017/S0007114525000911

**Published:** 2025-06-14

**Authors:** Shruti P. Shertukde, Marieke A.J. De Sévaux, Isabelle Herter-Aeberli, Tom Preston, Cornelia U. Loechl

**Affiliations:** 1 Nutritional and Health-Related Environmental Studies Section, Human Health Division, Department of Nuclear Sciences and Applications, International Atomic Energy Agency (IAEA), Vienna, Austria; 2 Global Health Unit, Department of Health Sciences, University Medical Center Groningen, Groningen, The Netherlands; 3 Population Research Centre, Faculty of Spatial Sciences, University of Groningen, Groningen, The Netherlands; 4 Institute of Food, Nutrition, and Health, ETH Zurich, Zurich, Switzerland; 5 Scottish Universities Environmental Research Centre (SUERC), University of Glasgow, Glasgow, UK

**Keywords:** Sustainable food systems, Stable isotope techniques, Protein digestibility, Iron balance, Breast milk intake

## Abstract

Global food security challenges, driven by the need to feed an estimated 10 billion people by 2050, require sustainable agricultural practices which strengthen nutritional adequacy while minimising environmental impacts. Yet, decision-making to foster food systems which consider both human and planetary health is growing in complexity. This paper, presented at an International Atomic Energy Agency-supported symposium at the 14th European Nutrition Conference of the Federation of European Nutrition Societies, highlights the potential of stable isotope techniques in generating valuable evidence to help support the development of sustainable food systems. It focuses on three methods: the dual tracer stable isotope technique for measuring protein digestibility, the Fe isotope dilution technique for assessing Fe absorption, loss and balance and the deuterium oxide dose-to-mother technique for estimating breast milk intake. The dual tracer isotope method provides a minimally invasive assessment of protein quality from a diverse variety of crops and novel sources, supporting the transition towards environmentally conscious, protein-rich diets. The Fe isotope dilution technique can be used to address Fe requirements across different population groups and calculate Fe absorption from whole diets or after consuming crops designed to be nutritionally sufficient, thus guiding dietary guidelines and agricultural strategies. Finally, the deuterium oxide dose-to-mother technique provides precise data on breast milk intake, underscoring the role of breastfeeding in sustaining optimal infant nutrition and the conservation of resources. These methods have the ability to generate critical evidence to support policy development and food system innovations that prioritise human health and environmental preservation.

The growing challenge to ensure global food security in the coming decades – feeding nearly 10 billion people by the year 2050 – would require a 60 to 70 % increase in food production over the next 30 years^(1–3)^. This demand for greater agricultural productivity and crop yield will heavily rely on natural resources that are already considered scarce, degraded and vulnerable within a rapidly changing climate^(3,4)^. Even so, patterns of consumption have shifted to include more resource-intensive animal-sourced foods (ASF)^(5)^. Despite its nutritional benefits, this dietary shift has led to unintended consequences for our food systems, particularly in terms of climate impact. Livestock production, along with its associated greenhouse gas emissions, continue to disproportionately affect biodiversity, land cover and resource availability^(6)^.

As such, the current ‘call to action’ for mitigating the impacts of agriculture and livestock rearing on food systems and climate change is supporting a global dietary transition towards ‘sustainable diets,’ or those diets which prioritise both human and planetary health^(7,8)^. More broadly, this can be achieved through increasing the intake of plant-based foods, lowering the consumption of ASF and minimising the degradation of natural resources^(8,9)^. Yet, consideration must be given to the immense variability in global agricultural practices and food consumption^(7)^, and the dietary needs of the most vulnerable – infants, children and older adults – who may heavily rely on minimally available, nutrient-rich foods (such as ASF) for growth, development as well as physical and mental function^(10)^. Such notions suggest that the pathways of dietary transition should differ based on contexts. For example, high and upper middle-income contexts, who consume the greatest proportion of ASF^(11)^, could benefit from replacing some ASF for plant-based alternatives.

Agriculture itself sits at the centre of food systems as its practices are directly responsible for producing the foods, and essential nutrients, for optimal nourishment. Food systems are also intimately linked to overall human health as the foods they supply provide the nutrients necessary to maintain the nutritional wellbeing of individuals and populations^(12)^. Thus far, however, agricultural practices, research and policy primarily focus on trying to increase crop yields, with minimal attention given to improving the nutritional quality of foods^(12)^. While increasing yields has been crucial in feeding growing populations and has proven to address protein-energy malnutrition deficits^(13)^, neglecting to provide nutritionally adequate crops will likely result in incidental deficits in various micronutrients^(14)^.

At the same time, there exists a transition in the diets of infants and young children to contain some, if not all, resource-intensive commercial milk formula (CMF) in place of breast milk^(15–17)^. The health benefits of breastfeeding are well-documented and include shaping immune function^(18)^ and preventing malnutrition, infectious and non-communicable diseases as well as mortality in infants^(19,20)^. Such benefits can also extend to mothers^(21,22)^ and involve broader economic implications^(23)^.

Despite the strong evidence and recommendation for exclusive breastfeeding during the first 6 months of life and continued breastfeeding up to 2 years of age, along with the significant progress made towards achieving the global exclusive breastfeeding target, fewer than half of infants under 6 months of age are exclusively breastfed worldwide^(24)^. Concurrently, the availability of CMF and its introduction to infant’s diets has grown^(25)^, creating significant challenges not only for exclusive breastfeeding, but also for food systems from the perspective of climate change. Compared to breastfeeding, CMF production requires the use of natural resources and generates considerable waste^(26)^. Thus, supporting breastfeeding not only promotes infant health, but also conserves resources and fosters more sustainable consumption patterns.

Decision–making to support optimal dietary transitions for both human and planetary health is becoming increasingly complex. This creates a growing need for precise and accurate tools to characterise the quality of specific dietary transitions – such as increased consumption of nutrient-rich plant-based foods and nutritionally adequate crops as well as enhanced breastfeeding – and assess how these transitions can not only improve nutrition outcomes but can also contribute to a more sustainable food system. Stable isotope techniques can be used to assess the nutritional value of foods, breastfeeding patterns and the impact of improved dietary quality on associated nutrition outcomes.

Thus, this paper aims to highlight the application of specific stable isotope techniques in assessing the nutritional value of foods and diets with respect to protein and Fe as well as characterising breastfeeding patterns. The information generated can provide valuable evidence to support the development of more sustainable food systems.

The stable isotope techniques discussed include:

- The dual tracer stable isotope technique for measuring protein digestibility;
- The Fe isotope dilution technique for assessing Fe absorption and loss;
- The deuterium oxide dose-to-mother technique for measuring breast milk intake.

The areas highlighted and the techniques discussed are described in more detail in Table 1 and were part of a symposium organised by the International Atomic Energy Agency (IAEA) and presented at the 14th European Nutrition Conference of the Federation of European Nutrition Societies in 2023, held in Belgrade, Serbia. During the symposium, experts and users of these techniques delivered presentations on the thematic areas outlined in this paper.

## Measuring protein quality from plant-based foods

Concerns over climate change highlight current food systems and agriculture as major contributors to greenhouse gas emissions^(27)^, with animal production generating significantly more emissions than plant production. This has led to recommendations for reducing the proportion of dietary protein derived from ASF, in particular. While there are various alternatives to animal protein, plant protein production already plays a significant role in agriculture. Compared to animal production, crop production generates far fewer greenhouse gases, uses less land, requires fewer agrochemical inputs and consumes less water^(28)^. Additionally, nitrogen-fixing legume crops, which are excellent sources of plant protein, have the unique ability to reduce the need for nitrogen-based fertilisers, a significant driver of emissions^(29)^. Yet, plant proteins are often viewed less favourably than animal proteins as they typically lack sufficient quantities of one or more of the nine indispensable amino acids (IAA) required for bodily function and growth^(30)^. However, most plant proteins are rarely consumed in isolation and are eaten in diverse mixed meals, where the amino acid deficiencies of one plant protein can be complemented by another. This raises protein quality by improving the amino acid score of the meal.

The importance of plant-based and novel foods (e.g. insects, algae, mycoproteins, etc.) lies in their potential to reduce environmental impact and improve human health. As scrutiny grows over the environmental costs of producing animal protein, there is an increased awareness of the lower environmental impacts of plant proteins. This has led to recommendations that plant proteins should make up a larger portion of total protein intake. With the shift towards reducing animal protein consumption – with such proteins described as being highly digestible and bioavailable – there is a need to understand the digestion of protein and the availability of amino acids from plant-based and novel foods to support their adoption as part of a sustainable diet.

Protein digestion is a complex process involving a cascade of enzymatic reactions and motility control in the gastrointestinal tract. Given this complexity, the FAO has recommended that protein digestibility should be assessed *in vivo*^(31)^. With time, extensive studies have been conducted in adults using intrinsic labelling of foods with ^15^N and collection of ileal samples, where the disappearance of amino acids at the terminal ileum serves as a measure of protein digestibility^(32–34)^. Stable isotope tracers are ideal for studying the rate and extent of absorption and metabolism *in vivo*, as there is no harm in their use for human studies. Some plant-based foods (e.g. legumes, cereals) have been grown in the presence of modest quantities of ^15^N, which labels the plant and its proteins uniformly. The disappearance of the labelled IAA from the intestinal lumen provides an accurate measure of digestion and absorption, unaffected by endogenous protein digestion or unlabelled proteins in a meal. However, the use of this method requires an invasive intubation procedure to collect ileal samples and is, therefore, unsuitable for children, older adults and those who do not wish to volunteer for such a procedure.

A dual tracer stable isotope technique was developed to eliminate the need for intubation by measuring protein digestibility through the appearance of IAA from a test protein in plasma, compared to the IAA appearance from a standard protein with a known digestibility^(35)^. The standard protein is also intrinsically labelled but with a different isotope. Spirulina whole cells, a highly digestible, commercially available ^13^C-labelled source of single-cell protein, are commonly used as the standard protein, but other standard proteins are also possible. Test proteins can be of plant, microbial or animal origin. Plant-derived test proteins are produced during plant growth, often under controlled environments, with the use of modest amounts of ^2^H_2_O or ^15^N-labelled fertilisers added to the soil to label IAA during seed development^(36)^. To avoid interference from transamination affecting the appearance of ^15^N-IAA in plasma, the method uses ^13^C for the standard protein and ^2^H for the test protein. As with ^15^N, such isotopes are also stable and are safe to use in human subjects. Both the test and standard proteins are incorporated into a standardised meal, with postprandial blood samples collected at multiple time points to quantify the isotopic ratios for each IAA. When the meal is taken in small, frequent boluses, the steady-state ratio of IAA enrichment of the test protein in plasma is compared to that of the standard protein using mass spectrometry^(35,36)^. A plateau feeding protocol was developed to minimise the number of blood samples required^(35)^.

The use of stable isotopes reduces the burden on study participants compared with invasive intubation methods but introduces greater complexity and costs to the laboratory phase. Measuring the enrichment of two isotopes currently requires separate mass spectrometers and trained personnel for sample collection, preparation and analysis. In addition, producing isotopically labelled proteins is an expensive undertaking, which may limit the accessibility of this method. Yet, the method itself has proven to be manageable in different countries^(37–39)^ and has even been used in studies involving young children^(40,41)^. Several crop varieties, including chickpea, mung bean, faba bean, yellow pea, kidney bean, pinto bean and rice, have been studied, consistently showing that plant-based foods may have lower protein digestibility than ASF such as egg, milk and meat. This lower digestibility is often attributed to factors such as reduced bioaccessibility, the presence of anti-nutritional compounds or amino acid modification during food processing. However, processing methods – such as de-hulling – can improve the digestibility of some plant-based foods^(42)^. Additionally, food preparation techniques – including extrusion – have been shown to enhance protein digestibility in plant-based foods comparable to that of ASF^(40)^.

There are numerous opportunities for the use of this stable isotope method. One key gap is the lack of information on protein digestibility from a wider range of economically significant crops, with a broader geographic representation. For instance, data on the protein digestibility of common leguminous crops from Africa are significantly underrepresented in existing studies. Expanding the diversity of crops studied could provide a more comprehensive understanding of not only protein digestibility but also of crops in different geographic areas, which could have significant environmental and economic potential as part of local sustainable food systems. As protein digestibility from plant-based foods is generally lower than ASF, it may be useful to explore the utility of additional processing and preparation methods to enhance the potential of plant-based alternatives^(43)^. Another area worth exploring is whether the bioavailability of other nutrients could be assessed within the same experiment. When crops have been intrinsically labelled with ^2^H_2_O (or ^13^CO_2_) as they grow, all organic molecules become labelled. As such, organic nutrients other than IAA, such as essential fats and vitamins, also become labelled. It may be beneficial to measure the availability of these other nutrients in conjunction with protein digestibility. Given the considerable effort and expense involved in conducting intrinsic labelling studies, measuring the bioavailability of multiple nutrients in a single experiment could offer value in understanding both the nutrient composition and overall bioavailability of plant-based foods and how essential nutrient supply may meet demand.

Taken together, the dual tracer stable isotope technique for measuring protein digestibility is a safe, minimally invasive method that is able to quantify the digestibility of IAA from plant-based and novel food sources in humans. This approach is suitable for use in vulnerable populations, including children, older adults and individuals with chronic diseases, to assess protein digestibility throughout the life cycle and under various health conditions. Additionally, this method has the potential to fill knowledge gaps with respect to understanding the protein digestibility from a diverse variety of crops grown globally as well as novel sources (including meat alternatives) and how various processing and preparation techniques can impact the protein digestibility of plants-based foods, all of which could inform how the diet may supply daily protein recommendations.

## Understanding iron absorption and loss from whole diets and interventions

Micronutrient deficiencies are often the result of inadequate intakes or excessive losses of vitamins or minerals^(44)^. They are also caused by malabsorption, which is commonly related to the presence of infection, inflammation or chronic disease^(45–47)^. Termed ‘hidden hunger,’ the occurrence of micronutrient deficiencies is typically less apparent than energy or protein malnutrition^(44)^. Yet, it is projected that over 2 billion people experience some form of micronutrient deficiency, far outweighing those affected by macronutrient malnutrition^(48)^. Micronutrient deficiencies can result in both minor and severe conditions, including the aggravation of diseases and infections, blindness, cognitive impairment, diminished growth and a general loss in human potential^(49,50)^.

Fe deficiency is reported to be the most common micronutrient deficiency^(51)^ and is closely related to the development of anaemia, where an estimated 50 % of all anaemia cases are caused by Fe deficiency, with certain variations between regions^(52)^. Fe deficiency, even without anaemia, manifests in a variety of symptoms including fatigue, lethargy, reduced concentration, dizziness, tinnitus, pallor and headache^(53)^. Even though it is acknowledged that a large proportion of anaemia is amenable to Fe supplementation, and thus due to Fe deficiency, population studies measuring Fe status biomarkers beyond haemoglobin are scarce. To a large extent, this is due to the complexity in interpreting those biomarkers, especially in conditions of clinical or sub-clinical inflammation^(53)^.

Conventional Fe status biomarkers – such as serum ferritin and soluble transferrin receptor – reflect Fe metabolism rather than Fe balance and thereby only indirectly inform Fe status. Furthermore, such biomarkers can be influenced by physiological factors, specifically infection and inflammation^(54)^. The Fe isotope dilution technique was therefore developed to directly determine Fe turnover, Fe balance, as well as the response to Fe interventions^(55–58)^. This methodology was based on earlier radioisotope studies, which applied a similar concept^(59,60)^.

The Fe isotope dilution technique relies on the fact that after equilibration, the concentration of the isotopic tracer in all body compartments is equal^(61)^. After tracer administration, a first enrichment is seen in red blood cells, which is then followed by a re-distribution of the tracer in the body after red blood cell senescence. It has been shown that equilibration is reached after approximately 12 months^(59)^, which can make this approach time-intensive, as it requires prolonged follow-up to ensure full equilibration as well as retention of the labelled Fe cohort. At this point, any Fe lost has the same isotopic composition as the total body Fe and will therefore not lead to a change in isotopic ratios. Thus, any change in isotopic tracer ratios or concentration can occur only by the addition of Fe with a natural isotopic composition in the form of Fe absorbed from the diet or supplements. In other words, a decrease in the concentration of the Fe isotope tracer in circulation is proportional to the amount of absorbed Fe. At the same time, a decrease in the absolute quantity of Fe isotope tracer can only occur if Fe, and thereby the tracer, is lost from the body. This concept can then be used to determine Fe balance, Fe absorption over time from whole diets, Fe losses, as well as the response to Fe interventions. Furthermore, the data can be used to accurately determine Fe requirements in different life stages.

This method has already been successfully applied in several studies to address Fe requirements across different population groups. In an early study by Fomon *et al.* (2003), Fe requirements were determined to be 1·46 mg/d for males and 1·15 mg/d for females, based on two measurements taken at around 13 and 17 years of age. Isotopes were administered at around 10 years of age as part of an Fe absorption study^(57)^. In a more recent study, Cai *et al.* (2018) determined the physiological Fe requirements of Chinese adults^(58)^. In this study, participants were given an intravenous dose of ^58^Fe and were followed for 1155 days. Participants were found to be fully equilibrated after 425 days, and the four blood samples collected thereafter, days 515, 605, 767, and 1155, were used to determine requirements. The estimated daily Fe requirements in this population were found to be 0·96 mg/d for males and 1·10 mg/d for females^(58)^. More recently, the method was used to determine Fe requirements in African children^(56)^. Children, recruited at an average age of 6·3 years after having participated in Fe absorption studies 5 years earlier, were followed for 2 years with blood samples taken every 3 months. The study confirmed that the WHO/FAO recommended Fe requirement of 32 µg/kg of body weight/day was adequate for Fe-sufficient children, but Fe-deficient children would need higher amounts to correct their deficit^(56)^.

The isotope dilution method has also been applied in determining Fe balance and the amount of Fe absorbed from whole diets or supplements. In a study by Speich *et al.* (2021), Fe balance was assessed in women from Switzerland and Benin before, during and after an Fe intervention, with each period lasting 3 to 4 months. The study revealed differences between the two population groups in terms of both Fe balance and the bioavailability of dietary Fe^(55)^.

The long equilibration period limits the use of this method for infants under 1 year of age unless they are already born in equilibrium. This was demonstrated in a recent study in which mothers were labelled with stable Fe isotopes during pregnancy, thereby resulting in their infants being born equilibrated^(62)^. By analysing blood samples collected at 2 days, 3 months and 6 months after birth, it was possible to calculate the amount of Fe absorbed and gained during this period based on feeding practices (breastfed, formula fed or mixed feeding). The data also allowed the determination of Fe bioavailability from the three different diets^(62)^.

The direct approach of measuring Fe absorbed, Fe lost, and thereby Fe gained, rather than using surrogate measures of Fe metabolism, opens doors for further applications in the future. One area of growing interest is the calculation of Fe absorption from whole diets. Traditional Fe absorption studies using stable isotopes typically focus on individual meals or specific Fe compounds, which do not reflect long-term Fe absorption from a certain diet. Other methods to estimate bioavailability from whole diets, such as algorithms based on intake data, have also faced challenges in practical application^(54,63)^. By directly determining Fe absorption and losses over extended periods of time, the isotope dilution technique can overcome these issues and allow for a more accurate estimate of Fe absorption from a given diet.

**Table 1.** Overview of the stable isotope techniques used to support sustainable food systems

Method	Measures	Methodology
Dual tracer stable isotope technique^(36)^	Protein and amino acid digestibility of foods	Involves the simultaneous ingestion of two stable isotopically labelled proteins – intrinsically labelled test (^2^H or ^15^N) and standard (^13^C) proteins – added to a standardised meal The test protein is generated by incorporating ^2^H or ^15^N into plant, microbial or animal-based proteins during biosynthesis. Isotopically labelled plant proteins are produced by growing plants in the presence of deuterated water (²H_2_O) or ^15^N-labeled fertilisers, ensuring uniform protein labelling The standard protein should be a highly digestible protein whose true indispensable amino acid (IAA) digestibility is known. A standard protein commonly used is ^13^C-spirulina whole cells In a plateau-feeding protocol, postprandial blood samples are collected, and the true IAA digestibility is determined by comparing the steady-state plasma enrichment ratio of IAA from the test protein to that of the standard protein using mass spectrometry
Iron isotope dilution technique^(84)^	Iron absorption and loss	A stable isotope tracer of iron (^57^Fe) is administered orally and becomes incorporated into erythrocytes. As these labelled erythrocytes reach the end of their life span, they break down, releasing the tracer into the circulating body iron pool, where it gradually distributes across all tissues After approximately 12 months in adults, or 8 months in infants and children, the tracer is uniformly enriched throughout the body’s iron stores. At this point, any iron with a natural isotopic composition (^56^Fe) that is consumed via diet or supplements will dilute the isotopically enriched body iron, so that the ^57^Fe concentration decreases. The rate at which ^57^Fe decreases in concentration is proportional to iron absorption Any iron that is lost has the same isotopic composition as the total body iron; thus, iron lost from the body will reduce the amount of tracer in the body. Blood samples are collected over a duration of time to capture the change in the slope – depending on the study design – and are analysed using ICP-MS or TIMS
Deuterium oxide dose-to-mother technique^(71)^	Volume or quantity of breast milk consumed	Before dosing, the mother should be weighed in light clothing to the nearest 0·1 kg, and the baby should be weighed naked to the nearest 0·01 kg. Baseline saliva samples are then collected from both the mother and baby Each mother receives a single oral dose of deuterated water (²H_2_O). The standard dose for assessing breast milk intake is 30 g at 99·8 at.% ²H, regardless of a mother’s body weight. After dosing, the mother should continue feeding her baby as usual, ensuring that the baby ingests ²H_2_O exclusively through breast milk Saliva samples are then collected from both the mother and baby at 1, 2, 3, 4, 13 and 14 days after the mother’s initial dose. Deuterium enrichment in the saliva samples is measured using FTIR, enabling the calculation of breast milk intake and the intake of water from sources other than breast milk

²H_2_O, deuterated water or deuterium oxide; IAA, indispensable amino acid; ICP-MS, inductively coupled plasma mass spectrometry; TIMS, thermal ionisation mass spectrometry; FTIR, Fourier-transform infrared spectroscopy.

With the aim to better understand the nutritional quality of crops and the nutrients they provide for human health, another approach could involve measuring total Fe bioavailability (e.g. Fe absorbed, lost and gained) after consuming crops designed to be nutritionally adequate, through biofortification or other methods. This approach would offer valuable insights as to how much Fe is absorbed, lost and retained by the body from crops cultivated either for higher yields or for enhanced nutritional quality. It could also increase our understanding of how efforts to improve food systems, such as through biofortification to increase the nutritional adequacy of crops, can impact Fe status and overall human health.

## Exploring the first food system: human milk intake

The first food system is broadly defined as the system that provides foods for children aged 0–36 months^(15)^. As the ‘first food’ for children, breast milk is a key component within this system. Breast milk provides optimal levels of nutrients and energy personalised to the needs of the infant by the mother–infant dyad^(64)^. For the first 6 months of life, breast milk alone suffices to meet the nutritional needs for growth and development, and it is a recommended staple food until 2 years of age^(64,65)^. The health benefits of breast milk and breastfeeding are clear for infants as well as mothers^(17,64,66)^.

However, the reality is that it is a global challenge to optimise breastfeeding rates^(19,64,67,68)^. Infant and young child feeding behaviours are shaped by numerous interlinked individual, political, social, cultural and economic determinants^(19)^. Although the influence or importance of these factors may vary among populations, breastfeeding is at risk in many countries due to the aggressive and inappropriate marketing of CMF^(15)^. The CMF industry undermines breastfeeding through inappropriate marketing that influences food environments and consumer behaviours^(69)^.

The threat CMF pose to breastfeeding is related to wealth^(15,64,70)^. Country-level data show that CMF sales follow a gradient across income levels, with sales being highest in high-income countries and lowest in low-income countries^(15)^. At the individual level, wealth (i.e. socio-economic status) is also related to breastfeeding, although the relationship varies depending on the context^(64,67,68,70)^. In high-income countries, breastfeeding is more common among those of higher socio-economic status, whereas the opposite is reported for low-income countries. The positive association of breastfeeding and socio-economic status in high-income countries may be indicative of a ‘new’ phase of returning to breastfeeding. At the same time, the rapidly growing CMF sales in middle-income countries suggest that these regions are in the middle of a dietary transition towards CMF, and it can be expected that low-income countries will follow soon if no action is taken.

It is therefore important to accurately monitor breast milk intake across countries and population groups to support breast milk intake as part of a sustainable food system. The deuterium oxide dose-to-mother technique – developed by W. A. Coward and colleagues in the UK in the early 1980s – is a precise, objective and safe tool to measure the average daily volume of breast milk consumed by infants^(71)^. It is an alternative to test weighing, where the infant’s weight difference before and after every breastfeed amounts to the volume consumed, a method that is imprecise, time-consuming and disturbs the normal feeding behaviours of mothers and infants^(71,72)^. The dose-to-mother technique requires the mother to consume a single oral dose of deuterated water, after which it mixes with her body water and a portion is transferred to the infant via breast milk. Deuterium oxide appears in the saliva or urine of mothers and infants and is repeatedly sampled over a period of 2 weeks, a sampling scheme and duration that could impact participant retention. Deuterium enrichment is then analysed using FTIR or mass spectrometry, enabling the calculation of water intake as human milk as well as water from sources other than milk. Besides the knowledge on the volumes consumed, information on both sources of intake additionally indicates whether breastfeeding was exclusive^(71)^. Such information cannot be captured using test weighing and is commonly measured through maternal recall, which has been shown to overestimate exclusive breastfeeding rates^(73)^. The dose-to-mother technique is therefore also a useful tool to evaluate policies and interventions aiming to protect, promote and support exclusive breastfeeding.

Diets high in CMF are not only harmful to infant and maternal health but also planetary health. CMF directly contributes to climate change through the resources needed for production, packaging, distribution and preparation, and the greenhouse gases emitted through these processes^(74,75)^. This is in stark contrast with the direct mother-to-child food chain, which only requires a marginal increase in energy intake for mothers (500 kcal/day when breastfeeding from birth until 6 months)^(76)^. Sustainability is therefore yet another argument in favour of breastfeeding and monitoring breast milk intake that deserves more recognition.

As of now, the first-food system is not included in the Conceptual Framework of Food Systems for Diets and Nutrition used in the United Nation’s Food Systems Summit^(77,78)^. It is important to acknowledge the significance of the first-food system by including it within the conceptual framework. A better understanding of breast milk intake, through the use of the dose-to-mother stable isotope technique, will be key to successfully attaining the Summit’s objectives in alignment with the sustainable development goals.

## Practical considerations and applications

A key consideration when using stable isotope techniques to inform sustainable food systems is the practical challenges associated with their implementation. Notably, all three of these techniques are more expensive than conventional methods and require instrumentation that is not widely available across laboratories globally and demand skilled personnel to perform the measurements. However, they enable the generation of accurate data that would be difficult, if not highly unlikely, to obtain in humans using other methods. Owino *et al.*^(79)^ emphasised the need to make stable isotope techniques more affordable, an aspect that remains crucial for their broader implementation. In addition, scaling up these methods in resource-limited settings is indeed difficult, but using the techniques to validate more field-friendly methods before their implementation is one way to scale up the assessment.

Despite these challenges, the evidence generated by these techniques has played a significant role in shaping practical applications which support sustainable food systems. For instance, the deuterium oxide dose-to-mother technique has provided valuable data verifying that exclusive breastfeeding for the first 6 months of life ensures infants receive sufficient energy for normal growth, thus supporting the WHO/UNICEF recommendation^(80,81)^. Moreover, a study using the dose-to-mother technique indicated that mothers living in food-insecure households often have limited breast milk output, which in turn leads to reduced caloric and micronutrient intake for their infants^(82)^. Such findings highlight the need for improved screening measures to identify at-risk mothers and provide targeted support to ensure both optimal and sustainable infant feeding practices.

In response to the growing concern about the environmental impact of heavily relying on ASF, the FAO and IAEA have initiated the development of a database on the protein quality of foods^(83)^. This resource, informed in part by stable isotope data, can help assess the long-term health effects of transitioning to more plant-based dietary proteins and aid in understanding the balance between meeting nutritional needs and maintaining planetary health. Data on protein digestibility and quality derived from stable isotope methods are already informing dietary recommendations for complementary feeding^(41)^. And as more data becomes available, the Fe isotope dilution technique also holds great promise to refine our understanding of Fe requirements in different contexts and inform the development of food programs or initiatives that meet these needs.

### Conclusions

Stable isotope techniques provide the rigor necessary to better understand the growing complexities in changing food systems. This paper discussed the current and potential application of three techniques in generating evidence to inform sustainable food systems. First, the dual tracer stable isotope technique was described as a safe, minimally invasive way to measure protein digestibility from plant-based and novel foods in humans, including vulnerable populations. This method has the ability to assess how various processing techniques affect digestibility and provides opportunities to study a wide variety of crops grown globally. Second, the Fe isotope dilution technique was identified as a direct method to measure Fe absorption, loss and balance in humans, offering insights into Fe requirements across different populations. This method can assess Fe bioavailability from crops and whole diets and aid in the evaluation of interventions that are designed to enhance the nutritional quality of foods. Finally, the first-food system, centred on breastfeeding, plays a critical role in infant nutrition and the conservation of resources. The deuterium oxide dose-to-mother technique provides a precise, non-invasive way to measure breast milk intake, supporting policies to aid in breastfeeding, ultimately benefitting both human and planetary health. As food systems continue to evolve, such techniques, among others, will be critical in generating evidence that ensures there are minimal negative impacts, and overall positive inputs, from these systems.
